# Tidal Wetland Soil Carbon Accumulation Rates for Coastal California

**DOI:** 10.1038/s41597-026-06935-8

**Published:** 2026-03-24

**Authors:** James R. Holmquist, Lauren N. Brown, Elizabeth Fard, Richard F. Ambrose, Kathryn E. Hargan, Douglas E. Hammond, Nathaniel J. Kemnitz, John P. Smol, Karen Thorne, Glen M. MacDonald

**Affiliations:** 1 Smithsonian Environmental Research Center, Edgewater, Maryland USA; 2 Bowling Green State University, Ohio, USA; 3 Environment and Climate Change Canada, Quebec, Canada; 4 University of California, Los Angeles, USA; 5 Memorial University of Newfoundland, St. John’s, Canada; 6 University of Southern California, Los Angeles, USA; 7 Fugro U.S.A. Marine, Houston, USA; 8 Queen’s University, Kingston, Ontario Canada; 9 U.S. Geological Survey, Western Ecological Research Center, Sacramento, California USA

**Keywords:** Carbon cycle, Ecosystem services, Climate-change mitigation

## Abstract

Carbon stock and carbon accumulation rate data are vital to multiple aspects of tidal wetland conservation and restoration policy. In California, USA tidal soil data are rare outside of the San Francisco Bay and Sacramento Delta regions, despite the differing conditions experienced by the outer coastline. Here we provide carbon stocks and decadal-to-centennial-scale carbon accumulation rate calculations. This dataset presents 83 soil depth profiles from 15 sites, with 58 cores from 12 tidal wetland sites analyzed for carbon stock, mostly from the outer coastline of California. Mean organic matter content was 11%, and stocks estimated to 1 meter depth ranged from 15.4 to 44.7 kgC m^−2^. Organic matter content generally declined asymptotically with depth. Carbon accumulation rates ranged from 39.2 to 130.0 gC m^−2^ yr^−1^. Neither carbon stock nor carbon accumulation rates were notably different from global average values. Data at this level of reporting are vital for establishing restoration baselines, informing greenhouse gas mitigation planning, and projecting future ecosystem response to sea-level rise.

## Background & Summary

Coastal ecosystems are unique in global change science because they form soil mass as a dynamic response to sea-level change^1–4^. In tidal wetlands, soils accrete via organic and inorganic pathways^5^, forming organic soil mass by subsurface root turnover^6^ and high rates of organic matter preservation^6,7^, and promoting mineral sediment layers by trapping suspended sediment associated with incoming tides^8^ and storm surges^9^. Both of these have some, but limited, capacity to increase with accelerating sea-level rise, causing a stabilizing feedback^10^. The burial of organic matter also causes a continuous sink, with necromass potentially stored on millennial scales, effectively sequestering carbon^1^.

Coastal wetland restoration, preservation, and enhancement are of interest to many parties, including land managers and policymakers, because of the potential for wetland conservation and interventions to improve carbon sequestration^11^, minimize methane emissions^12^, and enhance a suite of other ecosystem services^13^. Multiple projects on the U.S. Pacific Coast have incorporated large tidal reintroduction projects, such as in the Nisqually Delta in Washington State^14^ and the San Francisco Bay and Sacramento Delta of California^15^.

Projecting whether tidal wetlands will fail to keep pace with sea-level rise and convert to open water requires information about their accretion history, in tandem with process-based models^16^. Therefore, accretion, soil carbon, and bulk density information are vital to planning for conservation and management action to prevent loss. Data is required to plan interventions to stop the net losses of carbon stocks by strategies such as sediment augmentation^17^ and facilitating the upland migration^18,19^ of wetlands.

Tidal wetland restoration is a vital part of national and sub-national policies to offset climate impacts. Fargione *et al*.^20^ highlighted methane emission reduction from reintroducing saline tides to artificially freshened impoundments as a “Natural Climate Solution”. Importantly, carbon sequestration and methane emission reduction synergize with other co-benefits such as the conservation of habitats for endemic and endangered species, protection of coastal infrastructure from storm surges, improvements in water quality, and support for economically important fishery species^13^.

The State of California has a particular interest in managing coastal systems for carbon benefits. The State’s greenhouse gas reduction plan calls for a suite of carbon removal strategies, including natural carbon sequestration from protecting and restoring tidal wetlands^21^. A critical requirement for both supporting coastal restoration and conservation policies from the top down and management decisions from the bottom up is the curation of transparent disaggregated datasets for both carbon stocks and carbon accumulation rates^22^. A previous iteration of the State of California Natural and Working Lands Scoping Plan did not include tidal wetlands outside of the Sacramento Delta because data were limited^23^.

**Fig. 1:**
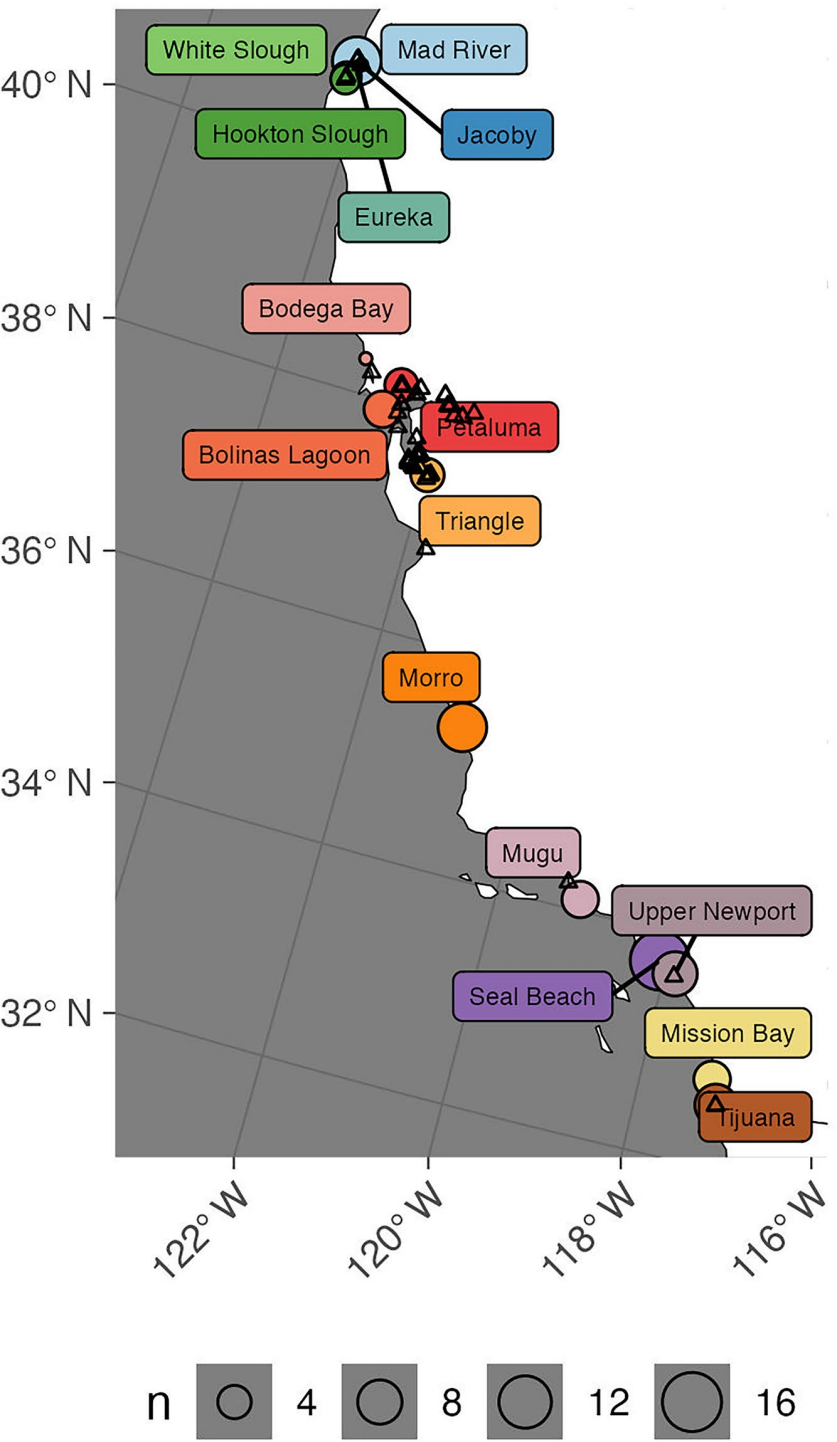
Map of the California, USA coast shows the latitudinal transect, with sampling sites labeled. The number of soil cores per location is indicated by the size of the point (n). Triangles indicate the locations of previous studies and sites.

Limited tidal carbon stock data exist for the outer coastline of California (Fig. 1). According to the Coastal Carbon Library^24,25^, there have been 15 previous studies published for California associated with 27 citations^4,15,26–50^. For example, Ward *et al*.^45^ sampled carbon stocks from seagrasses and tidal marshes from multiple sites in Southern, Central, and Northern California. Two studies have sampled tidal marshes in Humboldt Bay, Northern California^33,36^. Nahlik and Fennesey sampled multiple tidal wetlands within the state as part of the National Wetland Conditions Assessment^40^.

**Fig. 2:**
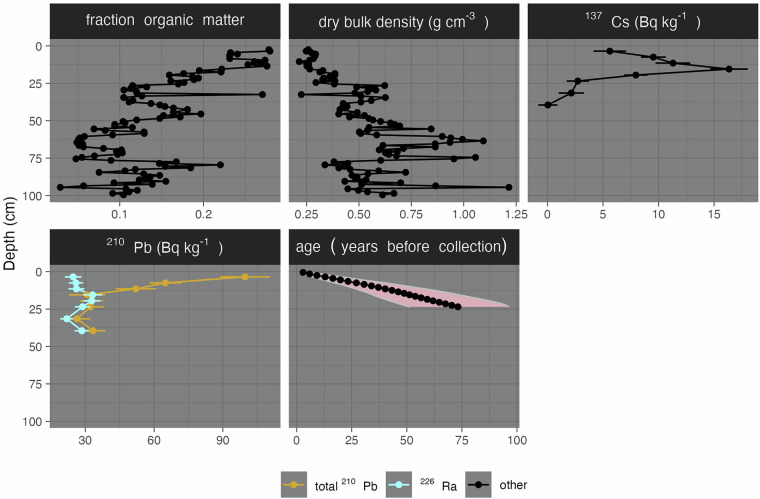
Example depth profiles from a single core from this dataset (SB15-11) including organic matter content, dry bulk density, a radiocesium peak indicating 1963 CE, and ^210^Pb, both total and supported (^226^Ra), as well as the age-depth model with propagated uncertainty.

As far as we know, current carbon accumulation rate data are almost entirely located in the San Francisco Bay and Sacramento Delta, and are absent from the Outer Coast in the literature. Currently available data for the San Francisco Bay and Sacramento Delta include ~50- to 100-year records of natural tidal marshes^4^, longer-term (centennial to millennial-scale) records of natural tidal marshes^47^, comparisons between restoration and reference wetlands^15,31^, and both nearer-^28^ and longer-term^51^ records of tidal freshwater forests, also known as swamps.

In this paper, we present a new dataset containing carbon stock estimates, radioisotope dating information, and accumulation rate estimates from 83 coastal marsh soil profiles from 15 wetland sites in California, mostly from the outer coastline. The tidal wetlands extend in latitude from near the Oregon border southward to the border with Mexico. We document the hierarchical, disaggregated data structure showing depth-series measurements nested within coring locations, nested within sites.

In addition to providing the dataset, we provide carbon stock calculations to a depth of 1 m and carbon accumulation rates. Finally, we provide R code demonstrating these calculations.

Earlier releases contained some of the information herein. Carbon stock data have been used in previous analyses^52,53^, but not published alongside the radioisotope dating information. Some radioisotope dating information was presented by Thorne *et al*.^18^ but is expanded upon here. Some radioisotope information was analyzed and presented in, but not published in disaggregated form, by Fard *et al*.^54^ (2021) and Brown^55^ (2019). This data release is the first attempt to synthesize carbon stock and carbon accumulation rate from a set of related studies taking place over the same time frame in California.

## Methods

### Field collection

This study presents data from a collection of 83 soil cores from 16 tidal wetland sites in California (Fig. 1). Cores were collected between 2012 and 2017. Five sites are located within Humboldt Bay, including White Slough (WTS), Mad River Low (MRL) and Mad River High (MRH), Jacoby Slough (JCB), Hookton Slough (HKS), and Eureka (ERK). Two additional sites were sampled on the outer coastlinenear San Francisco Bay, Bodega Bay (BOD) and Bolinas Lagoon (BOL). Two sites were within San Francisco Bay, Petaluma Marsh (PTL) and Triangle Marsh (TRM). The remaining sites were in central and southern California, including Morro Bay (MOB), Point Mugu (MGL), Seal Beach (SB), Upper Newport Bay (UNB), Mission Bay (MB), and Tijuana Estuary (TJE).

Cores were collected using a ‘Russian’- style peat corer, a meter-long half cylinder with a pivoting flap, that minimizes compaction. In the field, dominant plant species present at the sampling location were noted based on a visual inspection. General flooding zones were classified qualitatively based on known associations with vegetation species^56^. Latitude and longitude were recorded for each sampling point using a handheld GPS.

### Laboratory processing

Cores were transported intact as collected, usually in 1 m drives, to the University of California, Los Angeles. They were stored in plastic wrap to reduce moisture loss and refrigerated until processing. We subsectioned cores into 1 cm increments down to a depth of 1 m, and stored processed material in sealed plastic bags, which were refrigerated.

For each 1 cm depth increment, we measured organic content and dry bulk density. Dry bulk density was measured by allocating a standardized volume of 1 cm^−3^ of wet sample and drying samples in an oven set at 105 °C until constant mass was achieved. Organic content was measured using loss-on-ignition, the mass lost after 4 hours of ignition in a 550 °C muffle furnace^57^.

For select depth increments, we measured various radioisotope activities useful in radiometric dating techniques: radiocesium dating (^137^Cs), lead-210 (^210^Pb) dating, and radiocarbon (^14^C) dating. ^137^Cs and ^210^Pb dating were done at three different lab facilities: Core Scientific International, Queen’s University, and the University of Southern California. Activities of ^137^Cs, total ^210^Pb, and proxies for supported ^210^Pb (^226^Ra) were all measured using gamma detectors.

Material for ^14^C dating was recovered from select 1 cm subsamples and included plant and shell fragments. ^14^C dates were run at the Keck Lab at the University of California, Irvine. Because they use different calibration curves to convert radiocarbon ages to calendar ages, we note whether dates originate from carbonate or organic material alongside radiocarbon data.

### Data analysis

In analyzing the data, we estimated 1 m depth equivalent carbon stocks, created age-depth models from ^137^Cs and ^210^Pb profiles, and calculated the resulting carbon accumulation rates.

### Carbon stocks

Depth increment-level carbon density was estimated as the product of modeled organic carbon content and dry bulk density. We estimated organic carbon from loss-on-ignition by using a conversion function published by Craft *et al*.^58^ (Eq. 1).

1 $${OC}=0.40\,{LOI}+0.0025\,{{LOI}}^{2}$$

For each core, we summarized carbon stocks as the weighted average of the individual depth increments scaled to one meter depth. One meter depth is the default depth for existing global tidal carbon stock maps^59,60^ and the International Panel on Climate Change’s assumptions for the depth of carbon lost when wetlands are converted to open water or other land cover types^61^. Sensitivity analysis shows that the U.S. coastal wetland greenhouse gas inventory is highly sensitive to uncertainties in this assumption^62,63^.

**Fig. 3:**
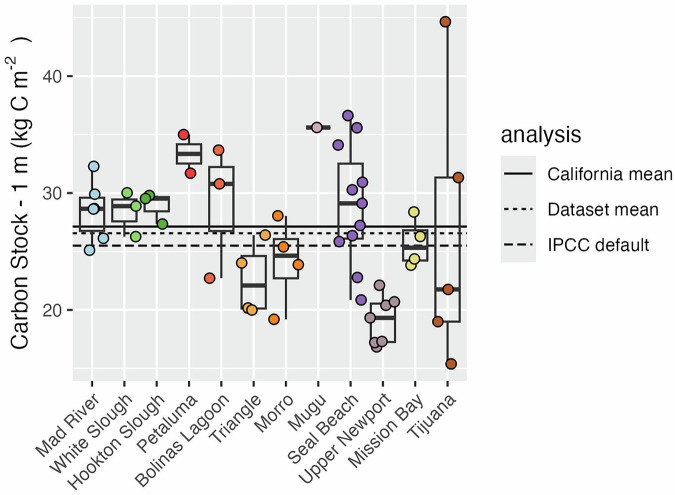
Carbon stock data for each core aggregated at 1 m depth. Sites are sorted by latitude; left to right indicates north to south. Each point represents an individual core, and bar and whisker plots represent site-level distributions. Central bars represent median values, box edges represent 25 and 75% quantiles, and whisker edges represent the most extreme values within 1.5 times the interquartile range. The total dataset’s mean value is displayed relative to the IPCC global default (Tier I) emissions factor for tidal marshes converted to open water, and the mean of 1 m integrated carbon stock estimates from previously measured locations in California.

We assessed 53 cores from 12 tidal wetland sites for carbon stocks that had a depth of at least 90 cm for carbon stocks (Fig. 3). In our dataset, mean carbon stock extrapolated to a depth of 1 m was 27.8 kgC m^−2^ (Fig. 3). There was little deviation between site level means and an IPCC default emission factor --the amount of carbon assumed lost per square meter when the top 1 meter of soil in a wetland is converted to open water --of 25.5 kgC m^−2^ for mineral and organic soils combined^61^. Average stocks were slightly lower than those estimated using the same method for a previous cores collected in California for tidal marshes (27.1 kgC m^−2^, n cores = 37)^27,32,36,40,43,47^.

Mean organic matter content in our new dataset was 11% organic matter by dry weight, slightly lower than the threshold above which soil bulk density becomes insensitive to further decreases in mineral content^52^.

**Fig. 4:**
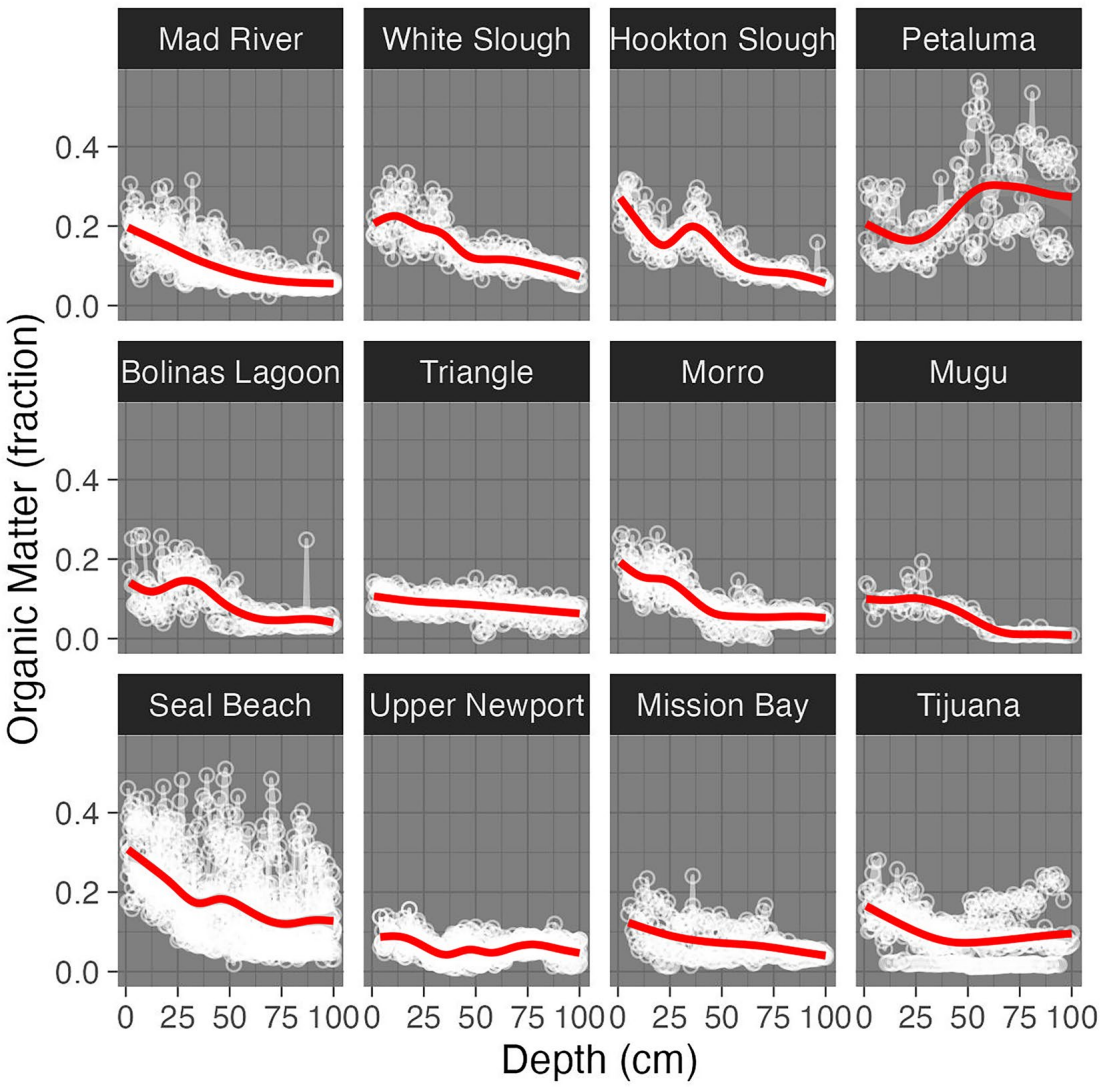
Relationships between depth and organic matter content for each core and each site. White points and lines indicate individual depth series. Red lines indicate smoothed site-wide trends.

For each core, we fit a linear model of organic matter content as a function of depth, using R^64^. Typical organic matter percentage in a core decreased with depth (Fig. 4). Average organic matter content was 19.5% for the top 5 cm of soil, which decreased to 8.1% at the 95 to 100 cm increment. Of the cores with at least 1 m depth, 82% had organic matter content that significantly decreased with depth, 9% had organic matter content that significantly increased with depth, and 9% displayed no significant depth-wise trends. Average slope for significant decreases indicated an organic matter loss of 0.13% per centimeter of depth.

### Carbon accumulation rates

In addition to carbon stocks, we provide decadal- to centennial-scale carbon accumulation rate calculations, comparing these new values to averages from existing data. To create decadal to centennial-length age-depth models, we used a combination of ^137^Cs and ^210^Pb dating.

In North America, peaks in ^137^Cs activity correspond to peaks in above-ground nuclear weapons testing ahead of the 1963 nuclear weapons test ban treaty^65^. However, a previous review by Drexler *et al*.^66^ showed that the decline of clarity of ^137^Cs peaks with time and geography results in decreasing confidence in the resulting accretion rate estimates. Therefore, we quantified the quality of these dates using three independent metrics and propagated uncertainty in the resulting accretion rates and age-depth models.

^137^Cs peaks were used only if they met three levels of automated statistical quality control. First, we determined whether peaks were significantly different from a background level, given the total distribution of ^137^Cs per core. We determined this using the *pnorm* function in R^64^ with the peak radioactivity as the value, the mean and standard deviation of the activity counts for all cores at the site, and a significance threshold of 97.5%. Second and third, we determined whether peaks were significantly greater than both their adjacent upper and lower samples, given radioactivity counting uncertainty. We calculated these as the proportional overlapping area shared by two normal distributions defined by each sample’s mean estimated radioactivity and counting uncertainty. We classified peaks as being significantly elevated compared to the shallower and deeper adjacent measurements if the difference in activity was more than 2 standard deviations. Pairs that shared less than 5% proportional area were considered significantly different.

Uncertainty in the ^137^Cs-based age was propagated by treating the age as a uniform distribution and substituting age for depth. We estimated the mean and standard deviation of the ^137^Cs age using a commonly available moment matching formula (Eqs. 2, 3)^67^.

2 $${\mu }_{A}=({A}_{{\max }}+{A}_{{\min }})/2$$

3 $${\sigma }_{A}=\sqrt{{({A}_{{\max }}-{A}_{{\min }})}^{2}/12}$$

${\mu }_{A}$ and ${\sigma }_{A}$ refer to the mean and standard deviation of the age. ${A}_{{\max }}$ and ${A}_{{\min }}$ refer to the minimum and maximum potential age given a uniform distribution.

We expanded the age-depth modeling and uncertainty propagation from the decadal scale to the centennial scale by combining ^137^Cs dates with ^210^Pb dating using *PLUM*^68^*. PLUM* is a Bayesian age-depth modeling algorithm based on the constant rate of supply (CRS) model^69^ that requires dry bulk density, a depth series of total ^210^Pb values, and associated radioactivity counting errors as input variables.

To prepare cores for PLUM dating, we interpolated any missing dry bulk density or loss-on-ignition values from neighboring values using the *approx* function in R^64^. We specified the year of core collection as the surface age.

The algorithm is flexible in that it can either estimate supported ^210^Pb from the depth series or use proxy measurements of ^226^Ra to calculate unsupported ^210^Pb. It can also integrate other types of dates as long as they have associated uncertainties. In the majority of cases, ^226^Ra was measured directly, and we specified that the algorithm assume radon profiles were variable. In one case, no radon was measured, so we assumed the deepest two increments established background unsupported ^210^Pb.

We set priors for *PLUM* to be informative enough to impose reasonable constraints on potential accretion and atmospheric ^210^Pb flux while allowing the profiles to determine the modeled accretion rates. Mean accretion was set to 2.86 years per cm and was informed from a review of ^210^Pb-based accretion rates^70^ that are independently held out from an existing disaggregated data synthesis^25^. We set the accretion prior to be lightly informative by setting the shape to 1. We set the memory prior using a 0.66 mean and strength of 10, indicating that any potential depth-wise autocorrelation is possible, but we assume it is more likely that we observe high autocorrelation given that tidal wetlands accrete over long periods due to changes in relative sea-level. We modeled atmospheric deposition of ^210^Pb using a global synthesis assembled by Zhang *et al*.^71^, using ordinary kriging^72,73^ to estimate the mean and standard error of log-transformed average annual ^210^Pb fallout at core locations. We then used the mean and a calculation of shape (mean/variance) as priors for ^210^Pb fallout. For the rest of the priors, we used *PLUM* generic inputs.

We calculated carbon accumulation rates from the carbon density depth profiles and *PLUM* age-depth models, using the raw Markov Chain Monte Carlo (MCMC) output of *PLUM* to propagate uncertainty in accretion. We discarded the initial 2,000 iterations of the MCMC and stored an additional 10,000 iterations. For each iteration of *PLUM* MCMC, we omitted all ages older than 100 years relative to the surface, assuming that the age-depth model becomes unreliable when older than that. For each MCMC iteration, we calculated carbon stock down to the bottom of the age-depth profile and divided carbon stock by the age of the bottom of the profile relative to the surface age.

We estimated the mean accretion rate of this dataset to be 3.40 mm yr^−1^. Accretion rates from individual cores ranged from 2.41 mm yr^−1^ to 3.97 mm yr^−1^ per year. In and south of San Francisco Bay, mean estimated accretion was higher than long-term relative sea-level rise over the instrumental period (2.22 mm yr^−1^ in San Diego and 1.98 mm yr^−1^ in San Francisco, CA)^74^. For three cores from Humboldt Bay, accretion was lower than relative sea-level rise over the instrumental period (5.04 mm yr^−1^ at North Spit, CA)^74^.

Mean uncertainty in accretion, expressed as a critical value (standard deviation/mean * 100%), was 17.4%. Uncertainty in the modeled accretion rate could arise from having too few dated samples, non-contiguous measurements, or an incompletely observed stock of supported ^210^Pb.

**Fig. 5:**
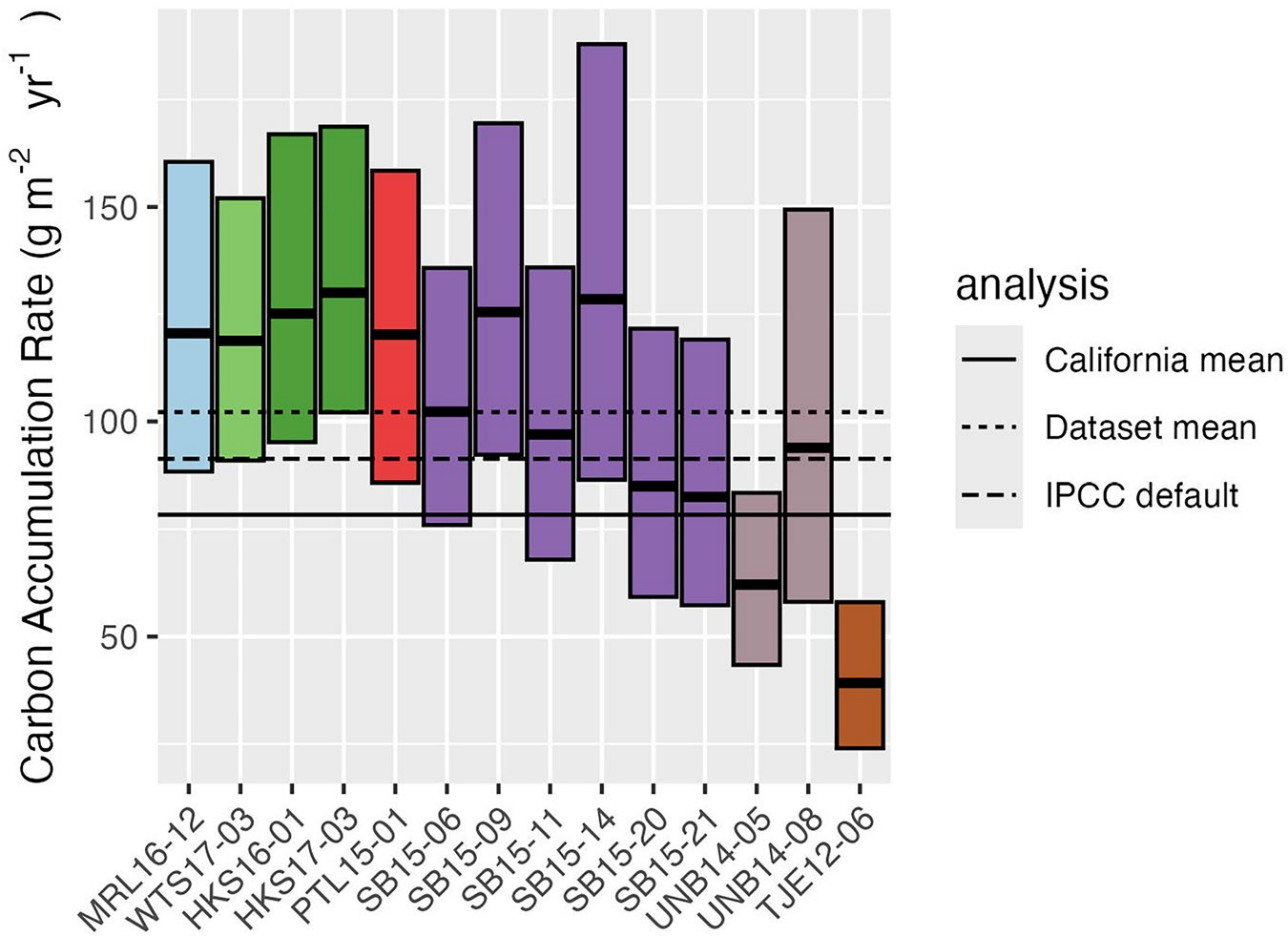
Carbon accumulation rates were calculated for a subset of 14 cores for which ^210^Pb and ^137^Cs profiles were present and passed all data quality control steps. We present mean and 95% credible intervals from probabilistic calculations, the dataset mean, the IPCC global default (Tier I) removals factor for tidal marshes remaining marshes, and the mean of reported ^210^Pb-based carbon accumulation rates from two previous studies from the San Francisco Bay region.

We estimate carbon accumulation rates to range from 39.2 to 130.0 gC m^−2^ yr^−1^ (Fig. 5). The mean estimate from our dataset (102.1 gC m^−2^ yr^−1^) is not markedly different from the default IPCC value for estimating carbon removals from rewetted tidal wetlands (91 gC m^−2^ yr^−1^)^61^, a figure which originates from a separate review of dated salt marsh sediment cores. It was slightly higher than carbon accumulation rates calculated from other ^210^Pb dated cores in the San Francisco Bay region of California (78.4 gC m^−2^ year^−1^) originating from 28 cores presented by Callaway *et al*.^4^ and three cores presented for Eden Landing by Arias-Ortiz *et al*.^15^ (2021). 95% credible intervals overlapped default IPCC removals factors for 9 out of 14 cores, and the California mean value for 6 out of 14 cores (Fig. 5).

## Data Records

The dataset presented is titled “Tidal Wetland Soil Carbon Accumulation Rates for Coastal California”^75^. It is hosted on Smithsonian Libraries’ FigShare and is formatted by modifying community-generated data entry templates and controlled vocabulary put forward by the Coastal Carbon Network^24^.

The data release contains a hierarchy of data related to wetland soil carbon profiles. The data itself is housed in four separate .csv files, which can be joined by the core_id and/or site_id attributes. Derivative calculations are kept separate from core and depth series tables and can be recreated with an R-code workflow.

- brown_et_al_2026_metadata - Definitions and units of attributes and variables within each table.
- brown_et_al_2026_materials_and_methods.csv - Contains information on materials and methods for this study, formatted so that it can be readily compared to others formatted using the same template.
- brown_et_al_2026_cores.csv - Positional and descriptive information on coring locations.
- brown_et_al_2026_depthseries.csv - Original measurements for the sampling intervals of each core.
- brown_et_al_2026_species.csv - Information on the dominant plant species at coring locations.
- brown_et_al_2026_site.csv - Site descriptive information.
- brown_et_al_2026_derivative_values.csv - Carbon stock and carbon accumulation rate information calculated from other tables.
- brown_et_al_2026_associated_publications.bib - Bibliographic information for publications associated with this data release.
- CA Pb 210 Eval.csv - Notes from visual inspection of ^210^Pb profiles.
- analysis - A directory containing a scripted workflow for analyzing carbon stock data, preparing derivative statistics, and creating data visualizations.

The dataset contains 83 cores and soil depth profiles, from 15 tidal wetland sites (Fig. 1). All sampling locations were naturally occurring, intact wetlands. None were receiving active management of water levels, nor had a significant history of extraction, diking, dredging, or replanting and restoration. Two tidal wetland sites occurred within the San Francisco Bay Region, and 14 were from coastal California. Collection years ranged from 2012 to 2017.

The final dataset contains 5,436 individual measurements of both dry bulk density and loss on ignition. 362 samples from 41 cores were measured for ^137^Cs activity, 327 samples from 38 cores contain total ^210^Pb measurements, most with the addition of supported ^210^Pb (^226^Ra), and 154 samples from 63 cores have been ^14^C dated.

We observed a range of modern-day ecohydrological conditions. 48% of cores do not specify a general flooding zone. However, 15.7% are specified as having originated from high-elevation marsh, 1% from high to mid elevation, 12% from mid elevation marsh, and 22.9% from low-elevation marsh. Six major plant species were dominant at coring locations, including *Salicornia pacifica*, *Distichlis spicata*, *Spartina foliosa*, *Spartina alterniflora*, *Frankenia grandifolia*, and *Jaumea carnosa*.

## Technical Validation

To quality control the dataset, we used a mixture of data visualization and automated statistical testing and filtering. First, we performed automated outlier filtering for loss-on-ignition by cores, omitting points that fall 1.5 times the interquartile range higher or lower than the 75th and 25th percentiles for a core (Fig. 2). Data visualizations that aided in detecting and correcting data entry errors included histograms of loss-on-ignition and dry bulk density, depth profiles of bulk density, loss on ignition, and radioisotopes (Fig. 2). We also visualized the relationship between loss-on-ignition and dry bulk density as it is well-constrained for tidal wetland soils^5^.

We performed both statistical and visual assessments of the data before calculating accretion rates. For ^137^Cs dates, we omitted any profiles that did not meet all three criteria under the scoring discussed in section 2.4. Of the 41 cores dated for ^137^Cs, 10 had statistically significant peaks representing 1963. For ^210^Pb data, we evaluated depth profile data using a methodology review by Arias-Ortiz *et al*.^76^ as a guide. We omitted any profiles that were likely to be indicative of past disturbance, that were ‘step-shaped’ or had ‘low activity’ for both ^210^Pb and ^226^Ra. These profiles are listed in a file in the workflow titled ‘CA Pb 210 Eval.csv’. Fourteen cores were classified as having profiles indicative of undisturbed conditions matching the assumptions of the CRS model.

## Usage Notes

It is important to note that the carbon accumulation rates presented here are not directly equivalent to the net ecosystem carbon balances. Our calculation of carbon accumulation rates ignores down-core trends in decomposition. It is probably more appropriate to refer to this measure as a long-term apparent rates of carbon accumulation (LARCA) as opposed to true rates of carbon accumulation, a distinction that is made more often in the peatland literature^77^.

The observed apparent decrease in organic matter content with depth could be representative of two possible scenarios, each of which could affect overall projections of the strength of the tidal wetland carbon sink in opposite ways. First, the downcore decrease in organic content could be due to decomposition of organic matter during burial. If this is the case, then this would suggest that LARCA overestimates net ecosystem carbon balance because the observed carbon accumulation incorporates both slowly decaying organic matter, which is effectively removed from the system over centennial scale time scales, as well as organic matter pools such as live roots and faster decaying organic matter, which will be returned back to the atmosphere relatively quickly. Second, alternatively, the apparent downcore decrease in organic matter content could reflect a recent increase in organic matter burial. Some studies indicate that accelerating sea-level rise may also be increasing carbon stocks^44^ and net ecosystem carbon balance^78^. If the downcore decrease in organic matter content reflects a recent increase in organic matter burial, then LARCA could be underestimating the strength of the tidal wetland carbon sink.

Caveats aside, these results offer a first-order assessment of carbon burial rate, and future studies could integrate core data with process models to bridge the theoretical gap between these observations and carbon cycle processes. The dataset presented here has utility for estimating baseline carbon stocks and accumulation rates. Uses include developing scenarios for tidal wetland restoration projects^15^, inventorying carbon fluxes on managed lands^79,80^, and fitting models of soil carbon accretion to forecast future wetland elevation under rising sea levels^16,81^.

## Data Availability

Both disaggregated and derived data presented in this paper is available in Brown *et al*.^75^, at the following link (10.25573/serc.28672772). Data is published under a Creative Commons (BY-4) license.

## Code availability

The code used to calculate carbon stocks, accretion rates, and carbon accumulation rates, as well as generate figures, is available in Brown *et al*.^75^, at the following link (10.25573/serc.28672772). Code is published under a Creative Commons (BY-4) license.
